# *Celosia argentea*: Towards a Sustainable Betalain Source—A Critical Review and Future Prospects

**DOI:** 10.3390/plants14131940

**Published:** 2025-06-24

**Authors:** Preekamol Klanrit, Sudarat Thanonkeo, Poramaporn Klanrit, Poramate Klanrit, Kanchanok Mueangnak, Pornthap Thanonkeo

**Affiliations:** 1 Department of Biotechnology, Faculty of Technology, Khon Kaen University, Khon Kaen 40002, Thailand; kpreek@kku.ac.th (P.K.); khanchanok.m@gmail.com (K.M.); 2 Fermentation Research Center for Value Added Agricultural Products (FerVAAP), Khon Kaen University, Khon Kaen 40002, Thailand; 3 Walai Rukhavej Botanical Research Institute (WRBRI), Mahasarakham University, Maha Sarakham 44150, Thailand; sudarat.t@msu.ac.th; 4 Research Group of Chronic Inflammatory Oral Diseases and Systemic Diseases Associated with Oral Health, Department of Oral Biomedical Sciences, Faculty of Dentistry, Khon Kaen University, Khon Kaen 40002, Thailand; porakla@kku.ac.th; 5 Department of System Biosciences and Computational Medicine, Faculty of Medicine, Khon Kaen University, Khon Kaen 40002, Thailand; porakl@kku.ac.th

**Keywords:** betalain, biological property, *Celosia argentea*, natural pigment, ornamental plant, plant secondary metabolites

## Abstract

Betalains are nitrogen-containing, water-soluble, and non-toxic natural pigments found in various plant species. Among these, *Celosia argentea* (Amaranthaceae) has garnered attention as a significant source, accumulating substantial quantities of both red–purple betacyanins and yellow–orange betaxanthins. Impressively, betalain concentrations in *C. argentea* inflorescences can reach up to 14.91 mg/g dry weight (DW), a level comparable to that reported in red beetroot. Beyond harvesting from inflorescences, betalains can also be produced using cell culture systems, which can yield even higher amounts, up to 42.08 mg/g DW. Beyond their role as vibrant natural colorants, betalains exhibit impressive health-promoting properties, most notably potent antioxidant activities. For instance, *C. argentea* inflorescence extracts demonstrate approximately 84.07% 2,2′-azino-bis(3-ethylbenzothiazoline-6-sulfonic acid) (ABTS) and 88.70% 2,2-diphenyl-1-picrylhydrazyl (DPPH) radical scavenging. Extracts derived from cell cultures show even higher scavenging capacities, reaching up to 99.28% for ABTS and 99.63% for DPPH, rivaling the antioxidant standard (ascorbic acid). Further research indicates additional potential benefits, including anti-inflammatory, antimicrobial, anticancer, antidiabetic, and hepatoprotective properties. This diverse bioactivity underpins their value across various industries. Betalains serve as natural colorants and functional ingredients in food and beverages, offer sustainable alternatives for textile dyeing, and hold therapeutic promise in cosmetics and pharmaceuticals. This review critically examines existing research on betalain production in *C. argentea*. Recognizing that research specific to *C. argentea* is less extensive compared with that on species such as *Beta vulgaris* and *Hylocereus polyrhizus*, this review analyzes its biosynthetic pathways, diverse biological properties, and wide-ranging applications. This is achieved by integrating available *C. argentea*-specific data with relevant insights drawn from these more broadly studied betalain sources. Furthermore, the review discusses perspectives on future research directions aimed at optimizing yield and exploring the full potential of betalains, specifically within *C. argentea*.

## 1. Introduction

**Figure 1:**
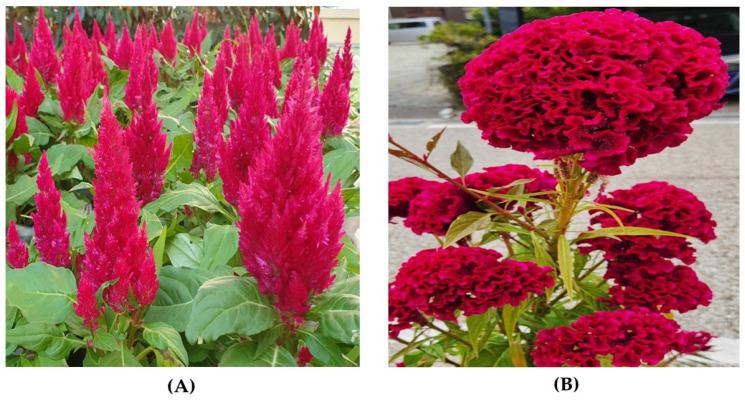
*Celosia argentea* var. *plumosa* (**A**) and *C. argentea* var. *cristata* (**B**).

The genus *Celosia*, belonging to the Amaranthaceae family, encompasses approximately 60 species native to subtropical and temperate regions of Africa, South America, and Southeast Asia [1,2,3]. The name “*Celosia*”, from the Greek “kelos” (burned), refers to its vibrant inflorescences. Among its species, *Celosia argentea* L. (considered the wild form by Miguel et al. [4]), along with its cultivated varieties such as *C. argentea* var. *cristata* (L.) Kuntze (cockscomb) and *C. argentea* var. *plumosa* (Burm.) Voss (feathered amaranth) are particularly recognized for their strikingly vibrant inflorescences (Figure 1), which contribute significantly to their ornamental value [5,6,7]. Beyond its aesthetic appeal, *C. argentea* also has a rich history of traditional nutritional and medicinal applications across various cultures [1,2,3,5,8,9,10,11,12,13].

The rich phytochemistry of *C. argentea* has been documented, revealing a diverse array of bioactive compounds such as saponins, polyphenols, fatty acids, peptides, amino acids, and minerals, among others [4,9,10,14,15,16,17,18,19,20,21,22,23,24,25,26,27,28,29]. These constituents contribute to a wide spectrum of reported biological activities, including antioxidant, antimicrobial, and anti-inflammatory effects [9,14,16,17,30,31,32,33,34,35]. A summary of these major bioactive constituents is provided in Table 1 (modified from Tang et al. [5]), underscoring the plant’s chemical diversity.

Numerous broad reviews have covered the phytochemistry and ethnopharmacology of *C. argentea* [5,10,14,16,18,19,20,21,22,23,24,25,26,27,28,29,31,36,37]. The literature has also addressed betalains across the wider Amaranthaceae family. However, a specific, up-to-date, and critical synthesis of *C. argentea* as a dedicated, potentially sustainable source of betalain pigments is notably less common compared with other betalain-producing plants. Despite this gap, the available literature indicates that betalains have been isolated and identified from *C. argentea*. Sources include inflorescences of both wild and cultivated plants, as well as callus and cell suspension cultures. For instance, Sidique et al. [38] reported betalain contents from *C. argentea* inflorescences ranging from 4.46 to 7.02 mg/g dry weight (DW). In contrast, Showkat et al. [11] reported a betalain yield of 1.40 mg/g DW. Another study by Mueangnak et al. [37] found substantial quantities of total betalains (2.95 mg/g DW) from 3-month-old field-cultivated inflorescences. Regarding cell culture systems, Guadarrama-Flores et al. [39] identified up to ten pigments. Notable betalains in their callus cultures included miraxanthin V (1.84 mg/g DW), vulgaxanthin I (2.01 mg/g DW), and betanidin (6.27 mg/g DW). Furthermore, they reported a high dopamine concentration of 42.08 mg/g DW in suspension cultures. This amount significantly exceeded the 19.62 mg/g DW found in the red callus line. More recent studies by Sang A Roon et al. [36] and Mueangnak et al. [37] also reported substantial betalain quantities—up to 4.99 mg/g DW—in cell suspension cultures of this plant. These nitrogen-containing pigments are gaining considerable scientific interest, arising from their potent biological properties, including antioxidant, anti-inflammatory, and anticancer activities [37,40,41]. These properties position them as valuable natural colorants and health-promoting agents.

Interest in natural pigments and sustainable resources is growing. Recent advancements have also occurred in betalain production using cell culture systems. Therefore, this review aims to critically assess and comprehensively describe the current research landscape of betalain production, specifically from *C. argentea*. The discussion addresses the betalain biosynthesis pathway within this species. Coverage extends to their quantified presence, biological properties, and diverse applications. Furthermore, this review synthesizes current knowledge to identify research gaps. It also explores future prospects for optimizing and scaling up betalain production from this promising plant resource. This focus distinguishes our work from broader phytochemical surveys or general reviews on betalains.

## 2. Literature Search Strategy

A comprehensive literature search was conducted to identify relevant studies for this review, with searches finalized in April 2025. The search primarily utilized the Scopus and Google Scholar electronic databases. The strategy employed a combination of keywords such as “*Celosia argentea*”, “betalain”, “Amaranthaceae”, “biosynthesis”, “phytochemistry”, “cell culture”, “bioactivity”, “antioxidant”, “application”, “commercialization”, “sustainability”, and other relevant synonyms or related terms. These keywords were used individually and in various combinations (e.g., “*C. argentea*” AND “betalain”; “*C. argentea*” AND “betalain” AND “phytochemistry”) to maximize the retrieval of pertinent literature.

An initial broad search for the term “betalain” across these databases yielded approximately 18,100 records. To specifically target literature relevant to this review, the search was refined by combining “betalain” with “*Celosia argentea*” and related terms. This more focused search strategy, after the removal of duplicate entries, resulted in 356 unique records. These records were then screened based on titles and abstracts against the predefined inclusion criteria: (1) focus on betalains from *Celosia* species, particularly *C. argentea*; (2) investigation of the phytochemistry, biosynthesis pathway, biological activity, or application of *Celosia* betalains; (3) status as peer-reviewed original research articles, review articles, or relevant book chapters; and (4) publication in English. Following this initial screening, the full texts of potentially eligible articles were assessed against these inclusion criteria and the exclusion criteria: (1) non-English articles where a reliable translation was not readily feasible; (2) conference abstracts, posters, or unpublished theses without accompanying full-text peer-reviewed papers; or (3) studies primarily focused on agronomic aspects without significant discussion of betalain chemistry or application. This systematic screening process of the 356 records yielded 38 articles that met all criteria for inclusion from the database searches, with Google Scholar being the more comprehensive source for these records.

To ensure comprehensive coverage and to capture foundational or highly specific studies that may not have been retrieved through the keyword-based database search, an extensive manual search of reference lists from the selected articles and other relevant reviews was conducted. This manual screening identified an additional 74 publications that met the inclusion criteria. Ultimately, a total of 112 studies were included in this comprehensive review.

## 3. *Celosia argentea* as a Source of Betalains

*C. argentea* flowers exhibit characteristic vibrant colors like red, orange, pink, and yellow, primarily attributed to the accumulation of betalains, a class of nitrogen-containing pigments. These betalains predominantly consist of red–purple betacyanins, including amaranthin, isoamaranthin, celosianin I, and celosianin II, alongside yellow–orange betaxanthins. Crucially, the precise composition and concentration of these pigments show significant variation depending on factors such as genotype, specific plant part, and prevailing cultivation conditions [1,36,37]. To facilitate direct comparisons where source data allows, betalain contents in this review are consistently reported on a fresh weight (FW) basis unless explicitly stated otherwise. This approach is also used when original studies only provided DW data without sufficient information for accurate conversion. For instance, illustrating genotypic differences based on FW, the total betalain content in *C. argentea* var. *cristata* ranges from 92.1 to 396.0 nmol/g FW. In contrast, *C. argentea* var. *plumosa* demonstrates a range from 456.5 to 621.5 nmol/g FW. Furthermore, variation by plant part is evident, as leaves reportedly contain higher betalain content (FW basis) compared to the epidermal layers of the stem [1].

**Figure 2:**
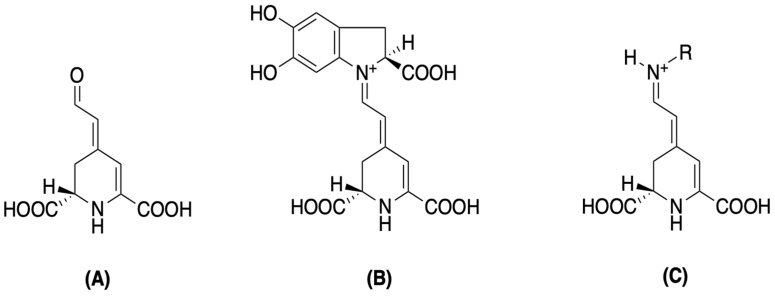
Betalamic acid (**A**), betacyanin (**B**), and betaxanthin (**C**) structures [46].

Structurally, betalains are defined as water-soluble, non-toxic plant secondary metabolites that are biosynthetically derived from L-tyrosine. They are broadly categorized into betacyanins, which exhibit a maximum absorbance of around 530 nm, and betaxanthins, with a maximum absorbance near 480 nm. Both groups share a common chromophore, betalamic acid [4-(2-oxoethylindene)-1,2,3,4-tetrahydropyridine-2,6-dicarboxylic acid]. This key structural unit is formed through condensation with either cyclo-3,4-dihydroxyphenylalanine (*cyclo*-DOPA) derivatives for betacyanins, or various amino acids/amines for betaxanthins (Figure 2) [42,43,44]. This inherent structural diversity, with over a hundred distinct betalains having been identified and categorized, results in the diverse colorations observed across various plant organs [42,45,46,47,48,49]. The betacyanin group includes compounds like betanin and amaranthin, while the betaxanthin group features various amino acid or amine conjugates [45,46]. Notably, when compared with anthocyanins, betalains often exhibit superior water solubility, greater dyeing capacity, and enhanced stability across a pH range of 3 to 7 [42,50]. These characteristics make them highly versatile for numerous applications.

**Figure 3:**
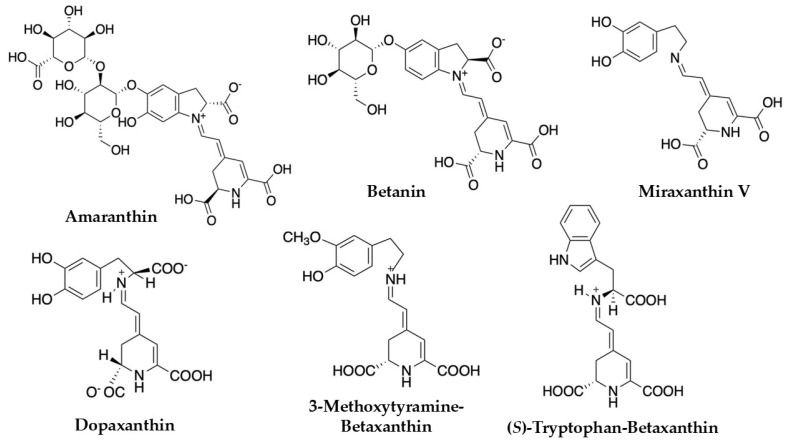
Betalains identified in *Celosia argentea* [1,55].

Within *C. argentea* specifically, investigations of plant inflorescences have led to the successful isolation and characterization of several important betalain compounds. These include prominent pigments such as amaranthin, betanin, betalamic acid, and various betaxanthins like miraxanthin V and dopaxanthin, and other betacyanins [1,36,37,51,52]. Representative structures of these compounds are illustrated in Figure 3 for clarity. Further research focusing on *C. argentea* cell cultures has revealed an even more extensive and complex profile of these valuable pigments. For instance, a comprehensive study by Lystvan et al. [53] on cell cultures of *C. argentea* var. *cristata* identified a diverse array of compounds. Among the betaxanthins reported were γ-aminobutyric acid-betaxanthin, indicaxanthin, miraxanthin V, valine-betaxanthin, phenylalanine-betaxanthin, and tryptophan-betaxanthin. The identified betacyanins in the same study included amaranthin, isoamaranthin, betanin, celoscristatin, isobetanin, and several malonylated derivatives like 4′-O-malonyl-amaranthin and phyllocactin. Complementing these findings, Mastuti et al. [54] detected several betalain compounds from callus cultures of *C. argentea*, such as amaranthin, isoamaranthin, betalamic acid, miraxanthin V, and (S)-tryptophan betaxanthine. The identification of such a broad spectrum of compounds, from both whole plant inflorescences and sophisticated cell culture systems, underscores the significant potential of *C. argentea* as a rich and varied source of betalains.

It is important to acknowledge that while the broader class of betalains is extensive, with over seventy structures previously characterized [42], our understanding specific to *C. argentea* has key limitations. A truly comprehensive picture of its complete betalain profile is still in the process of developing through ongoing research efforts. Furthermore, the intricate factors governing betalain biosynthesis and their subsequent accumulation within this particular species have not yet been fully elucidated. Similarly, a consolidated view of their stability under various processing and storage conditions remains a critical area requiring further investigation. Addressing these existing knowledge gaps is crucial to fully harnessing the potential of *C. argentea* as a robust and specialized source of these bioactive pigments. 

## 4. Biosynthesis Pathway of Betalains in *C. argentea*

Betalain biosynthesis in *C. argentea* involves intricate biological and chemical reactions, and the complete pathway specific to this species remains to be fully elucidated. Existing knowledge is largely extrapolated from research on other betalain-producing plants, such as *Beta vulgaris*, *Portulaca grandiflora*, *Mirabilis jalapa*, *Hylocereus undatus*, *H. monacanthus*, and *Amaranthus tricolor* [43,44,56,57]. Nevertheless, drawing from comprehensive literature reviews and evidence proposed by Schliemann et al. [1], a foundational understanding exists: betalains are synthesized from the aromatic amino acid tyrosine, which originates from the shikimic acid pathway [58]. These pigments are produced within the cytosol and endoplasmic reticulum before being transported to and accumulated in vacuoles, predominantly in epidermal and subepidermal tissues [59,60].

**Figure 4:**
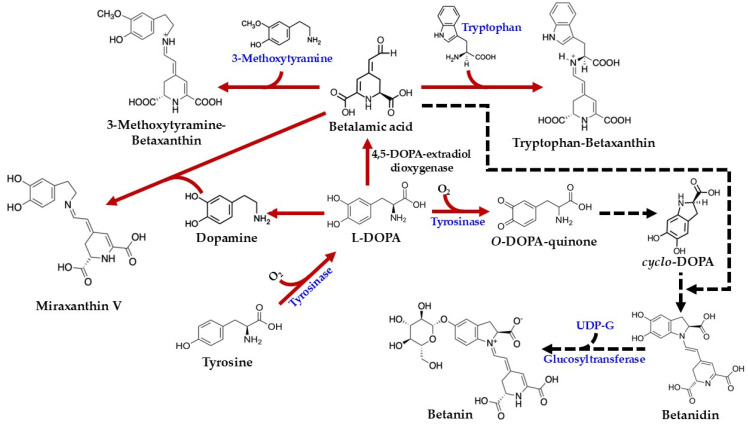
Biosynthesis pathways of betalains in *C. argentea* (adapted from [1,4,46]). Arrows indicate process steps: confirmed steps are shown as solid red arrows, and inferred steps are shown as dashed black arrows.

As illustrated in Figure 4, the biosynthesis of betalains commences with the conversion of tyrosine to 3,4-dihydroxy-L-phenylalanine (L-DOPA). While this initial step was previously attributed to tyrosinase activity in the presence of molecular oxygen [45,48], more recent studies have demonstrated that cytochrome P450 enzymes of the CYP76AD family are responsible for this tyrosine hydroxylation [61]. Subsequently, the aromatic ring of L-DOPA undergoes oxidation and cleavage, catalyzed by 4,5-DOPA-extradiol-dioxygenase (4,5-DODA), to yield 4,5-seco-DOPA [62]. This product, 4,5-seco-DOPA, then spontaneously cyclizes to form betalamic acid [63]. Betalamic acid is a crucial intermediate, serving as the precursor for both betacyanins and betaxanthins. The formation of betaxanthins is a relatively straightforward, non-enzymatic process involving the condensation of betalamic acid with various amino acids or other amines [42]. For example, condensation with tryptophan yields tryptophan-betaxanthin, condensation with 3-methoxytyramine produces 3-methoxytyramine-betaxanthin, and condensation with dopamine results in miraxanthin V.

In contrast, the formation of betacyanins follows a more intricate pathway, though specific information regarding their biosynthesis in *C. argentea* is limited. The process begins with the conversion of L-DOPA to *o*-DOPA-quinone, a reaction catalyzed by tyrosinase in the presence of molecular oxygen. Subsequently, a spontaneous cyclization occurs, involving the amine group of the *o*-quinone, to form *cyclo*-DOPA. This *cyclo*-DOPA then reacts with betalamic acid to generate the pigment betanidin [4].

Betanidin can undergo further modification. For instance, its glycosylation at position 5, catalyzed by betanidin-5-*O*-glucosyltransferase, results in the formation of betanin [4]. An alternative route to betanin has also been proposed: *cyclo*-DOPA itself can be glycosylated by *cyclo*-DOPA-5-*O*-glucosyltransferase to yield *cyclo*-DOPA-glucoside. The condensation of this glycosylated intermediate with betalamic acid then forms betanin [64,65]. Betanin, a prominent member of the betacyanin group, is characterized by a hydroxy group (–OH) at the C6 position and a glycosyl residue linked to the –OH at the C5 position. The synthesis of other betacyanins involves additional structural modifications, typically catalyzed by various glucosyltransferase enzymes. For example, gomphrenin features a glucosyl residue linked to the –OH at the C6 position. Amaranthin, similarly, contains a glucuronyl glucosyl residue at the C6 position. Bougainvillein is distinguished by having glucosyl residues at both the C5 and C6 positions of the original *cyclo*-DOPA moiety [4,49].

## 5. Production of Betalains from *C. argentea*

*C. argentea* is a highly versatile herbaceous plant cultivated worldwide, serving not only as an ornamental species but also as a food source and traditional medicine in regions including Asia, Africa, and South America [5,8,9,10]. Due to its dual cultivation as both a leafy vegetable and an ornamental, comprehensive global production data distinguishing these uses is lacking. To evaluate its potential as a sustainable source of betalains, this review collates available literature on its key agronomic proxies and compares them with established crops. Table 2 provides a structured comparison of *C. argentea* against major betalain sources: beetroot and dragon fruit.

As shown in Table 2, the reported biomass yields for *C. argentea* are comparable to established crops. However, these yields exhibit significant variability, ranging from 6.8 to 39.0 t/ha. This wide range reflects agronomic factors like plant density, fertilizer, and harvesting time [66,67,68]. These yields fall within the spectrum of beetroot (15.0–62.3 t/ha) and dragon fruit (2.97–41.55 t/ha), suggesting strong production potential [69,70,71,72]. Regarding betalain content, *C. argentea* inflorescences are a particularly potent source. However, the final yield is highly dependent on the extraction methodology employed. For instance, concentrations range from 1.40 mg/g DW with water extraction [11] to an impressive 14.91 mg/g DW using aqueous ethanol [73]. This peak value is competitive with and can exceed many beetroot extracts (2.40–12.60 mg/g DW) [75,76]. In contrast, it falls within the broad range reported for dragon fruit, which can reach exceptionally high levels (1.40–35.12 mg/g DW) [78,79]. In terms of productivity, data suggest that field-cultivated *C. argentea* (0.03 mg/g·day) is comparable to beetroot [37]. It may even be superior to dragon fruit under certain conditions [80]. This evidence demonstrates that *C. argentea* is a powerful and promising alternative pigment source.

However, Table 2 also highlights a critical gap in its sustainability profile. Crucial data on its water requirements are currently absent (‘n.a.’). While its biomass and betalain yields are high, its sustainability cannot be fully assessed. This is especially true when compared to drought-tolerant sources like dragon fruit. This data gap means the claim of *C. argentea* as a “sustainable source” remains partially speculative. Future agronomic research must therefore quantify its life-cycle inputs, particularly water usage and carbon footprint.

**Figure 5:**
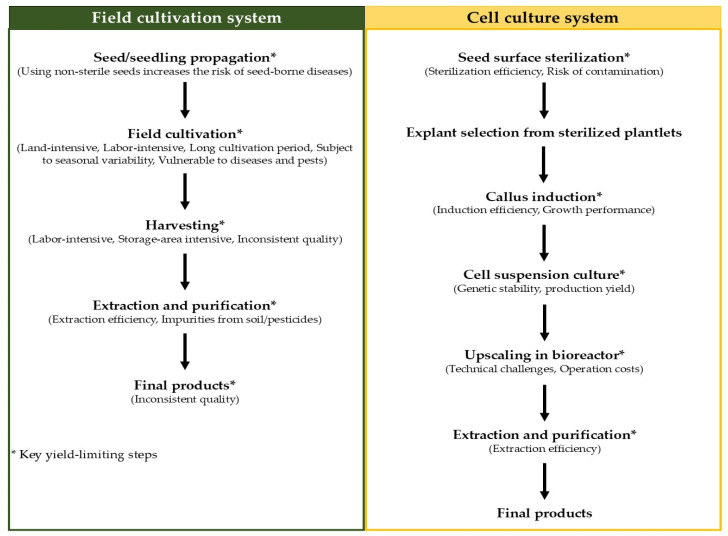
Diagram comparing the field cultivation and cell culture systems for betalain production from *C. argentea*.

Although betalains can be produced from wild or field-cultivated *C. argentea*, with the plant’s inflorescences serving as particularly rich sources, this traditional production system presents several significant challenges. Firstly, the process is notably labor-intensive, requires extensive land area, and is highly vulnerable to seasonal, climatic, and geographical variations. Secondly, the susceptibility of these plants to pathogens can significantly reduce betalain yield. Crucially, the productivity of betalain from wild or field-cultivated plants is relatively low; Mueangnak et al. [37] reported a betalain productivity of only 0.03 mg/g·day for field-cultivated *C. argentea*. These constraints have prompted researchers to explore alternative production techniques, with plant cell and tissue cultures emerging as promising platforms for the efficient synthesis of these valuable bioactive compounds. A simplified diagram comparing the field cultivation and cell culture systems, highlighting the key yield-limiting steps for each, is presented in Figure 5. Compared with traditional production, this platform overcomes the limitations of field-cultivated plants and provides a stable, economical, and sustainable supply of products.

Despite the potential of *C. argentea*, research into its betalain production using plant biotechnological methods remains relatively limited compared with other plant sources. Early investigations include the work of Guadarrama-Flores et al. [39], who successfully established callus and cell suspension cultures of *C. argentea* var. *plumosa*. They identified up to ten pigments, with notable betalains in the callus, including miraxanthin V (1.84 mg/g DW), vulgaxanthin I (2.01 mg/g DW), and betanidin (6.27 mg/g DW). Furthermore, their cell suspension cultures exhibited higher levels of these compounds compared with callus cultures. Interestingly, they also reported a high dopamine concentration of 42.08 mg/g DW in suspension cultures, significantly exceeding the 19.62 mg/g DW found in the red callus line.

Research on *C. argentea* var. *cristata* has also yielded significant findings. Warhade and Badere [51] investigated betalain production from its callus cultures, documenting substantial betalain content (calculated from amaranthin, betanin, betalamic acid, and betaxanthin levels) ranging from 9.62 to 29.90 mg/g FW. Building on this, the same researchers later explored the effects of various elicitors on betalain production in cell suspension cultures derived from this variety, finding that fungal elicitation resulted in a total betalain content of approximately 1.44 mg/g FW [52]. Further elaborating on *C. argentea* var. *cristata* callus cultures, Mastuti et al. [54] reported the production of miraxanthin V (0.23–0.46 mg/g FW), isoamaranthin (0.25–0.68 mg/g FW), and amaranthin (0.36–0.78 mg/g FW).

More recent advancements continue to demonstrate the potential of this approach. Sang A Roon et al. [36] established a betalain-producing cell line from *C. argentea* var. *plumosa*, achieving a maximum betalain concentration of 2.16 mg/g DW by cultivating cells in MS medium supplemented with 43.88 g/L sucrose, 0.15 mg/L tyrosine, and 0.77 mg/L BAP. Following this, Mueangnak et al. [37] investigated the impact of biotic and abiotic elicitation on betalain production in cell suspension cultures of *C. argentea* var. *plumosa*. Their findings revealed that chitosan (5.0 mg/L) and copper sulfate (CuSO_4_ at 6.4 mM) were highly effective elicitors, enhancing betalain production to impressive concentrations of 4.65 and 4.99 mg/g DW, respectively (Table 3).

A direct comparison using Table 3 reveals a pivotal conclusion. Optimized cell culture systems for *C. argentea* can outperform traditional field cultivation in both betalain content and productivity. Data show that the maximum betalain content from cell cultures significantly surpasses yields from field-grown inflorescences. Furthermore, Table 3 highlights that their productivity is also demonstrably higher. This rate of synthesis far exceeds the slower accumulation process seen in whole plants. This dual advantage fundamentally validates the pursuit of biotechnological methods. It shifts the paradigm from merely creating an alternative to establishing a superior production platform. This ability to achieve higher yields and faster production makes cell culture exceptionally promising. It offers a pathway to a reliable, scalable, and commercially viable betalain source. This method also operates independently of the climatic and geographical constraints tied to agriculture. Consequently, future research should focus on process engineering and bioreactor optimization to harness this proven potential at an industrial scale.

## 6. Biological Properties of *C. argentea* Betalains

**Figure 6:**
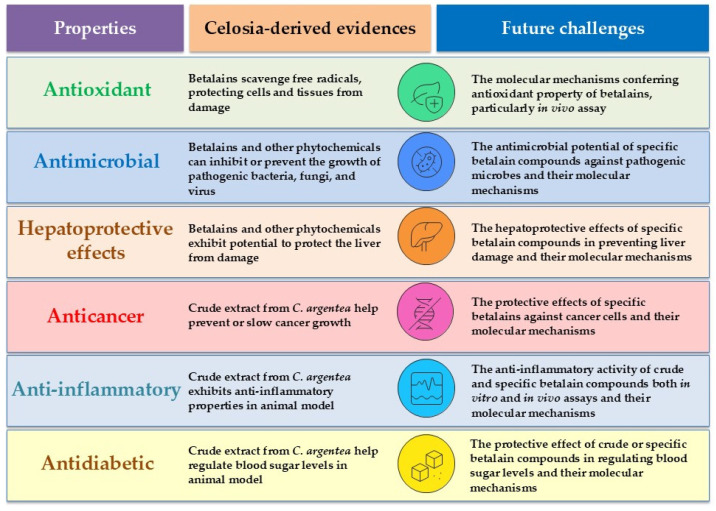
Biological properties of *C. argentea* betalains and their phytochemicals.

Generally, betalains exhibit a wide spectrum of biological activities, as demonstrated in both in vitro and cellular assays. These activities include antioxidant, anti-inflammatory, antimicrobial, anticancer, antidiabetic, antilipidemic, and hepatoprotective effects (Figure 6). However, beetroot and prickly pear (*Opuntia ficus-indica*) are primary sources for betalains in biological property investigations. Betanin and indicaxanthin are the most extensively studied compounds in this significant pigment family [44]. Regarding the biological properties of *C. argentea* betalains specifically, available information in the literature is relatively limited. The information concerning the biological effects of *C. argentea* betalains can be summarized as follows:

### 6.1. Antioxidant Activity

The antioxidant potential of betalains derived from *C. argentea* is a prominently studied biological property. This significance underscores their importance as natural protective agents against various forms of oxidative damage. Early investigations by Cai et al. [81] provided foundational insights into these valuable compounds. They reported strong antioxidant activities for various betalain compounds isolated from this specific plant. Their assessment utilized the EC50 value as a metric for radical scavenging activity. This value is the concentration in µM required for a 50% decrease in 2,2-diphenyl-1-picrylhydrazyl (DPPH) radical absorbance. It revealed distinct potencies among different betalain types found within the plant material. For instance, celosianins, a group of amaranthin-type betacyanins from *C. argentea* var. *cristata*, exhibited an EC50 value of 7.13 µM. While this indicates notable activity, it is comparatively less potent than typical beetroot betalain EC50 values (4.88–4.89 µM). However, the same study highlighted that betaxanthin-type betalains extracted from *C. argentea* var. *plumosa* showed more potent results. Specifically, dopamine-betaxanthin and 3-methoxytyramine-betaxanthin demonstrated lower EC50 values of 4.08 µM and 4.21 µM, respectively. These findings underscore their superior radical scavenging capabilities, even surpassing those commonly reported for red beetroot betalains.

Furthermore, Cai et al. [81] elucidated crucial structure–activity relationships for these antioxidant compounds. They demonstrated that the free radical scavenging efficacy of these betalains is augmented by a higher number of hydroxyl and imino groups. These groups are located within the molecular structure of the betalain compounds. The presence of a hydroxyl group at the C-5 position on the aglycone moiety also significantly enhanced activity. Conversely, increased glycosylation of aglycones was observed to clearly diminish their overall antioxidant potential. This provides valuable chemo-structural insights for understanding their functional properties and potential applications.

Subsequent research has further expanded our understanding of the antioxidant capacities of these betalain-rich extracts. These extracts were sourced from *C. argentea* inflorescences, often employing different extraction systems. A broader array of analytical methodologies was also used in these more recent studies. Thiyajai and Koyama [73] systematically evaluated betalains extracted from red *C. argentea* inflorescences. They used a water–ethanol system for this specific extraction process. Their comprehensive analysis encompassed oxygen radical absorbance capacity (ORAC), ferric-reducing antioxidant power (FRAP), and DPPH radical scavenging assays. This analysis revealed a consistent trend: antioxidant activities exhibited a clear dependence on the ethanol concentration used in extraction. Notably, the ORAC values for extracts obtained with 60% and 80% aqueous ethanol surpassed ascorbic acid. Ascorbic acid is a widely recognized antioxidant standard used for comparison purposes.

However, this superior performance was not uniformly observed across all conducted antioxidant assays. The FRAP values and the IC50 determined by the DPPH assay indicated stronger antioxidant capabilities for ascorbic acid—this was when compared to the *C. argentea* extracts in those specific tests. This discrepancy led Thiyajai and Koyama [73] to infer a predominant mechanism of action. They drew upon the principles of the ORAC assay for their interpretation. This assay primarily quantifies the capacity to scavenge peroxyl radicals via a hydrogen atom transfer (HAT) mechanism. It does not rely on a single electron transfer (SET) mechanism [82,83]. They posited that *C. argentea* antioxidants likely function as effective chain-breaking antioxidants. This is particularly significant, given that peroxyl radicals are major contributors to lipid peroxidation. This process occurs within human biological systems and can cause significant cellular damage. The constant exposure of the human biological system to numerous free radicals is well-established. The resultant imbalance between these reactive species and antioxidant defenses is a factor in non-communicable diseases [72]. Thus, Thiyajai and Koyama’s [73] findings highlight the therapeutic potential of *C. argentea* extracts. These extracts could be useful in mitigating such detrimental radical-induced damage. Nevertheless, the authors appropriately emphasized the necessity for in vivo experiments to substantiate these findings. Such experiments would help better understand the bioavailability and physiological relevance of these compounds.

Corroborating the general antioxidant potential of this plant, Lavanya et al. [84] also reported promising activities. These antioxidant activities were from aqueous extracts of *C. argentea* inflorescences. Their study utilized a comprehensive suite of assays, including 2,2′-azino-bis(3-ethylbenzothiazoline-6-sulfonic acid) (ABTS) radical scavenging and DPPH. They also used FRAP, cupric ion-reducing antioxidant capacity (CUPRAC), and metal chelating potential tests. Although the antioxidant properties observed in their study were generally less potent than ascorbic acid. The collective evidence from these diverse studies consistently points towards *C. argentea* inflorescences. They are considered a valuable natural source of various antioxidant compounds.

Beyond the inflorescences, the antioxidant properties of betalains derived from *C. argentea* cell cultures have garnered attention. This presents a sustainable alternative for large-scale pigment production for various applications. As demonstrated by Sang A Roon et al. [36], betalains from cell suspension cultures exhibited robust ABTS scavenging activity. This activity ranged from 86.66% to 86.88%, which was notably high. These values notably exceeded that of the inflorescence extract (84.07%) in the same assay. However, in this particular assay, all *C. argentea* samples showed lower ABTS scavenging activity than ascorbic acid. In contrast, when assessed using the DPPH assay, all samples demonstrated similarly strong antioxidant capacity. This included those from cell suspension cultures (88.81–90.39%) and inflorescence extracts (88.70%).

Further reinforcing the potential of cell culture systems, Mueangnak et al. [37] demonstrated a strong correlation. This correlation was between antioxidant activity and total betalain content in *C. argentea* cell suspension culture extracts. The maximum antioxidant activities achieved were approximately 99.63% radical scavenging activity (DPPH assay). They also found 99.28% inhibition (ABTS assay), indicating very high efficacy. These figures were notably higher than those detected in plant inflorescences under their experimental conditions. These inflorescence values were 91.26% DPPH scavenging and 89.29% ABTS inhibition. Neither Sang A Roon et al. [36] nor Mueangnak et al. [37] definitively determined the precise antioxidant mechanisms. This was for betalains from cell culture systems, requiring further research. They proposed that the observed activity is likely due to the inherent ability of betalain structures. These structures can directly and effectively scavenge various types of free radicals. Additionally, they suggested an indirect mechanism involving the activation of endogenous antioxidant defense systems. These systems include antioxidant enzymes or extracellular signal-regulated kinase (ERK) pathways, as previously reviewed [44].

Collectively, these findings highlight the significant antioxidant potential of betalains from *C. argentea*. This is true whether sourced from inflorescences or advanced cell culture systems. The investigations into cell suspension cultures are particularly promising for future applications. They suggest an efficient and alternative platform for producing betalains with potentially enhanced antioxidant properties. This warrants further exploration for their diverse health-promoting applications in various fields.

### 6.2. Antimicrobial Activity

The antimicrobial potential of extracts derived from *C. argentea* has been documented in scientific literature, pointing towards its considerable utility in effectively combating various pathogenic microorganisms. However, a critical gap exists in specifically attributing these observed antimicrobial properties directly to the betalain constituents present within the plant material. *C. argentea* is recognized as a rich repository of diverse bioactive compounds, including phenolics, flavonoids, alkaloids, tannins, and steroids, among others. Consequently, observed antimicrobial activities are likely a result of the complex interplay or synergistic effects of these various phytochemicals, rather than solely the direct action of betalains.

Several studies have thoroughly investigated the broad-spectrum antimicrobial effects of extracts obtained from different parts of *C. argentea*, particularly its betalain-rich inflorescences. Yun et al. [85] demonstrated that extracts from the inflorescences of *C. argentea* var. *cristata*, a significant source of betalains, exhibited notable antimicrobial action against a panel of pathogenic microbes. This panel included the yeast *Candida albicans* and several bacteria such as *Staphylococcus aureus*, *Escherichia coli*, *Pseudomonas aeruginosa*, *Bacillus subtilis*, and *Salmonella typhimurium*. Utilizing the broth microdilution method, following CLSI guidelines M07-A7 for bacteria and M27-A2 for yeast, to determine Minimal Inhibitory Concentration (MIC) values. The authors found that both methanolic and ethanolic extracts were particularly effective, reporting MIC values of 0.125 mg/mL against *S. aureus*, 0.5 mg/mL against *B. subtilis*, and 1 mg/mL against *C. albicans*. In a complementary study, Sridevi et al. [86] reported that crude extracts from the floral petals of *C. argentea* var. *cristata* elicited antimicrobial activity against both Gram-positive and Gram-negative bacteria. The Gram-positive bacteria tested included *B. subtilis*, *S. aureus*, and *B. megaterium*, while Gram-negative bacteria included *Proteus vulgaris* and *E. coli*. Interestingly, among the bacteria tested, *B. subtilis* was found to be particularly susceptible to the crude extract, exhibiting an inhibition zone of 8 mm in agar diffusion assays.

Further corroborating these important findings, Rao et al. [87] investigated inflorescence extracts of *C. argentea* var. *cristata* and reported potent antimicrobial activity against a range of different bacteria. These bacteria included *B. subtilis*, *S. aureus*, *E. coli*, *Klebsiella pneumoniae*, *P. aeruginosa*, and *Proteus vulgaris*, as well as filamentous fungi such as *Aspergillus* sp., *Penicillium* sp., *Fusarium* sp., and *Rhizopus* sp. Their results, which were based on the zone of inhibition assays, showed bacterial inhibition zones ranging from 5.0 to 9.0 mm for the tested strains. More substantial inhibition zones, measuring 12.0 to 23.0 mm, were observed against the tested fungi, suggesting a pronounced antifungal capacity of the extracts.

The antimicrobial efficacy is not limited to inflorescences, as Subba and Basnet [88] found that an ethanol extract of the whole plant of *C. argentea* demonstrated promising antimicrobial activity. This activity was observed against tested microorganisms, including *S. aureus*, *K. pneumoniae*, *P. vulgaris*, and *E. coli*, indicating broad-spectrum potential. Expanding on this, Okpako and Ajibesin [89] illustrated that extracts from the leaves, stems, and roots of *C. argentea* all elicited antibacterial activity against a comprehensive panel of strains. These strains included *E. coli*, *P. aeruginosa*, *B. subtilis*, *S. aureus*, *C. albicans*, and *A. niger*, with zones of inhibition ranging between 7 mm and 26 mm. Notably, in their study, only the leaf extract exhibited antifungal activity, highlighting potential organ-specific distribution or concentration of these particular antifungal compounds. In a novel application, Shahzadi et al. [90] explored the use of whole plant extracts of *C. argentea* for the green synthesis of cobalt nanoparticles (CoNPs). These plant-mediated CoNPs were then tested for their antimicrobial efficacy, and the study illustrated significant antimicrobial activity against both Gram-negative and Gram-positive bacteria. Specifically, an inhibition zone of 51.83 mm was observed against *E. coli*, and a 42.18 mm zone against *B. subtilis* was reported. This information suggests that *C. argentea* extracts can serve as effective reducing and capping agents for synthesizing nanomaterials with enhanced biological activities.

While the precise molecular mechanisms conferring the antimicrobial activity of *C. argentea* extracts, particularly the specific contribution of betalains, remain less elucidated through current research. Inferences can be drawn from studies on other betalain-producing plants and from known antimicrobial phytochemicals found within plant matrices. Betalains, similar to phenolic and tannin compounds, may exert antimicrobial effects by targeting microbial cell membranes, potentially altering their function and structure. This could lead to increased membrane permeability and ultimately result in microbial cell death, effectively controlling pathogen growth. Other proposed mechanisms, as reviewed by Sharma and Sharma [91] and Carreón-Hidalgo et al. [44], include binding to microbial adhesins, thereby preventing attachment to host cells. They also involve inhibition of essential microbial enzymes, deprivation of substrates necessary for microbial growth, complexation with cell wall components leading to structural damage, direct membrane disruption, and complexation with metal ions crucial for microbial metabolism. The presence of multiple phytochemical classes in *C. argentea* suggests that its antimicrobial action likely involves a multifaceted attack on microbial cells, contributing to its overall efficacy.

Beyond antibacterial and antifungal effects, *C. argentea* extracts have also demonstrated significant antiviral potential against a range of different viral pathogens. Early work by Balasubrahmanyam et al. [92] and later by Begam et al. [93] identified antiviral proteins, specifically glycoproteins designated CCP-27 and CCP-25, in the leaf extracts of *C. argentea* var. *cristata*. These proteins were shown to possess both deoxyribonuclease (DNase) and ribonuclease (RNase) activities, which are crucial for their antiviral action. Their efficacy was demonstrated against Torula yeast rRNA and several plant viruses, including citrus ringspot virus, tobacco mosaic virus, and sunnhemp rosette virus. These findings indicate a potential role for *C. argentea* in plant virus management and possibly as a source of novel antiviral agents for broader applications.

### 6.3. Hepatoprotective Effect

Extracts from *C. argentea* have garnered considerable attention for their potential hepatoprotective effects, as documented in the existing scientific literature. However, a comprehensive understanding remains somewhat limited, particularly concerning the direct contribution of its betalain constituents to these observed benefits. This is in contrast to the collective action of its diverse phytochemical makeup, which includes many other bioactive compounds. This section reviews the existing evidence supporting the hepatoprotective activities of *C. argentea* extracts, focusing on studies that investigate its potential to mitigate liver damage and modulate liver-related pathological processes.

A significant body of evidence for the direct hepatoprotective action of *C. argentea* comes from the detailed work of Kim et al. [94]. They undertook a detailed evaluation of the antioxidant potential and protective effects of *C. argentea* var. *cristata* inflorescence extracts against tert-butyl hydroperoxide (t-BHP)-induced oxidative damage. This evaluation was conducted both in Chang liver cells in vitro and in rat livers in vivo, providing a comprehensive assessment. In the in vitro setting, Kim et al. [94] demonstrated that these extracts exerted a protective effect by virtue of their potent radical scavenging ability. This antioxidant action translated into enhanced cell viability, prevention of excessive reactive oxygen species (ROS) generation, and inhibition of mitochondrial membrane depolarization in Chang liver cells subjected to *t*-BHP-induced hepatotoxicity. Furthermore, the in vivo component of their study involved oral administration of the *C. argentea* extract (at doses of 100 mg and 500 mg/kg body weight) to rats. This administration occurred for five consecutive days prior to inducing liver damage with a single dose of *t*-BHP (2 mmol/kg, intraperitoneally). This pre-treatment regimen showed a statistically significant (*p* < 0.05) protective effect, evidenced by a reduction in serum levels of key liver injury markers, glutamate oxaloacetate transaminase (GOT) and glutamate pyruvate transaminase (GPT). The extract also mitigated oxidative stress by decreasing hepatic levels of malondialdehyde (MDA), an indicator of lipid peroxidation, and reducing serum triglyceride (TG) levels in the face of *t*-BHP-induced oxidative stress. These comprehensive results led Kim et al. [94] to conclude that *C. argentea* extract effectively prevented oxidative stress-induced liver injury by significantly bolstering the antioxidant capacities of hepatocytes.

While not directly examining protection against chemical-induced liver cell damage, another line of research highlights the potential of *C. argentea* in addressing other liver-related pathologies. Jo et al. [95] conducted a study aimed at identifying multi-functional medicinal herbs capable of inhibiting Hepatitis C Virus (HCV) replication, a major cause of liver disease. Among the herbs screened, the authors reported that the red flower extract of *C. argentea* var. *cristata* displayed a notable cholesterol adhesion effect, which is relevant to viral entry. More critically, among the six selected herbs, this flower extract significantly inhibited HCV genotype 2a RNA replication, indicating direct antiviral activity. Jo et al. [95] suggested that these findings position the red flower extract of *C. argentea* var. *cristata* as a promising candidate for developing therapeutic agents. These agents could be used to treat HCV infection and associated hypercholesterolemia, both of which can profoundly impact overall liver health and function.

Further supporting the pharmacological relevance of *C. argentea*, particularly its inflorescences, Singh et al. [96] demonstrated the broad pharmacological capabilities of methanolic extracts from *C. argentea* var. *cristata* inflorescences. Their phytochemical analysis revealed that the plant extracts contain a rich array of constituents, including alkaloids, tannins, carbohydrates, saponins, terpenoids, phenols, flavonoids, and steroids. Singh et al. [96] reported that these bioactive phytochemical compounds present in the methanolic extracts exhibited various pharmacological capabilities, explicitly including clearly defined hepatoprotective effects. This observation further suggests their potential application in pharmaceutical industries for the development of new drugs aimed at improving and maintaining liver health.

Collectively, these studies suggest that *C. argentea* extracts, particularly those derived from the inflorescences, possess significant and demonstrable hepatoprotective potential. This potential appears to be mediated, at least in part, by strong antioxidant mechanisms, as well as through influencing viral replication and lipid metabolism relevant to various liver diseases. However, as with the other biological activities of *C. argentea*, the specific contribution of betalains versus other co-existing phytochemicals to these hepatoprotective effects remains an area requiring more focused investigation. Future research should aim to isolate betalains and other defined compounds from *C. argentea* to systematically evaluate their individual hepatoprotective activities. It should also elucidate their precise mechanisms of action, thereby paving the way for a fuller understanding and potential therapeutic exploitation of this valuable plant resource.

### 6.4. Other Pharmacological Properties

Beyond its well-documented antioxidant, antimicrobial, and hepatoprotective capacities, extracts derived from various parts of *C. argentea* have demonstrated a remarkable spectrum of other pharmacological activities. These include anti-inflammatory, antidiabetic, antidiarrheal, and antitumor effects, underscoring the plant’s rich and diverse medicinal potential for various health applications. However, a common thread across many of these studies is that the specific contribution of betalains to these observed biological activities remains largely underexplored. Research often focuses on crude extracts or other classes of phytochemicals isolated from seeds, leaves, or the whole plant, rather than specific pigments. This section provides an overview of these diverse properties, including anti-inflammatory, antidiabetic, antidiarrheal, antitumor, and immunomodulatory activities attributed to the plant.

The anti-inflammatory potential of *C. argentea* has been a significant area of investigation, yielding promising results in various experimental models. Bhujbal et al. [17] explored the anti-inflammatory activity of an alcoholic extract obtained from *C. argentea* leaves using established animal models for inflammation assessment. Their research employed the carrageenan-induced rat paw edema model for acute inflammation and the cotton pellet-induced granuloma model for evaluating chronic inflammation responses. The authors reported that the leaf extract, which they identified as predominantly containing flavonoids, exhibited significant, dose-dependent anti-inflammatory properties in both of these validated models. This led Bhujbal et al. [17] to conclude that the flavonoids present in *C. argentea* leaves are primarily responsible for the observed anti-inflammatory effects demonstrated in their study. Further contributing to the understanding of anti-inflammatory compounds in this species, Wu et al. [20] isolated several triterpenoid saponins from the seeds of *C. argentea*. They specifically named these compounds celosin E, celosin F, celosin G, and cristatain, identifying their chemical structures. These isolated active constituents were subsequently screened for their anti-inflammatory activity using in vitro methodologies, confirming the role of saponins, in addition to flavonoids, in mediating the plant’s anti-inflammatory response.

The traditional use of *C. argentea* in managing diabetes has also found considerable support in modern scientific studies evaluating its efficacy. Vetrichelvan et al. [33] highlighted the utility of an alcoholic extract derived from *C. argentea* seeds for the effective treatment of diabetes mellitus. To validate this traditional claim, Vetrichelvan et al. [33] evaluated the anti-diabetic activity by meticulously monitoring blood glucose levels and body weight in alloxan-induced diabetic rats. Their findings indicated that continual administration of the seed extract over a period of two weeks significantly reduced blood glucose levels in these diabetic rats. Importantly, the treatment also demonstrated a protective effect against the characteristic body weight loss associated with alloxan-induced diabetes, suggesting a multifaceted beneficial impact in this metabolic disorder.

The efficacy of *C. argentea* extracts in managing diarrheal conditions has been thoroughly investigated by Sharma et al. [97], providing scientific validation. The authors evaluated the antidiarrheal effect of a *C. argentea* leaf extract using several experimental models: castor oil-induced diarrhea, the charcoal meal transit test, and prostaglandin E2 (PGE2)-induced diarrhea. Their results suggested that the leaf extract, administered at doses of 100 to 200 mg/kg, effectively inhibited diarrhea in the tested animal models. The authors posited that the extract might exert its antidiarrheal effects through a central mechanism and by inhibiting the synthesis or action of prostaglandins. Specifically, the extract demonstrated protection against PGE2-induced enteropooling, likely due to the inhibition of prostaglandin synthesis within the intestinal tract. Furthermore, it significantly decreased the propulsive movement in the charcoal meal study, with the 200 mg/kg dose proving more efficacious than the standard antidiarrheal drug, atropine (2 mg/kg), indicating potent antimotility and antisecretory properties.

A compelling body of research points towards *C. argentea* as a potent agent for tumor treatment, often linked to its notable immunomodulatory capabilities. Hayakawa et al. [34] investigated the anti-metastatic effect of extracts from *Semen Celosiae* (seeds of *C. argentea*) in preclinical cancer models. They found that intraperitoneal administration of the seed extract for seven days prior to tumor inoculation significantly inhibited liver metastasis. This inhibition was caused by the intraportal injection of colon 26-L5 carcinoma cells and occurred in a dose-dependent manner, showing increasing efficacy with higher doses. Their in vitro experiments further revealed that a water extract of *C. argentea* mediated macrophage activation, leading to the production of factors that could influence white blood cell behavior. This macrophage activation potentially contributes to an overall antitumor environment within the host. The antitumor foundation of *C. argentea* has been attributed to its immune regulatory characteristics, including the induction of crucial cytokines such as IL-12, IL-2, and IFN-γ. This induction results in an immune state skewed towards B-cell dominance and the activation of various immune cells to achieve an antitumor state. For instance, co-culture of celosian (a polysaccharide from *C. argentea*) with Concanavalin A (Con A, a T-cell mitogen) was shown to increase IFN-γ secretion twofold compared with Con A alone. This indicates that celosian not only activates macrophages but also significantly affects T-cell function, enhancing immune responses. Supporting these immunomodulatory effects, Devhare et al. [98] demonstrated the significant immunomodulating activity of extracts obtained from the aerial parts of *C. argentea*. They screened both 70% ethanol and water extracts for their effects on delayed-type hypersensitivity, neutrophil adhesion, and cyclophosphamide-induced myelosuppression. These tests were conducted in Swiss albino mice, providing further evidence of the plant’s capacity to effectively modulate diverse immune responses within a biological system.

In reports on the antitumor activity of *C. argentea*, triterpenoid saponins are frequently identified as key bioactive compounds responsible for these effects. Huang et al. [99] reported that celosin A—a specific triterpenoid saponin—was effective in inducing apoptosis in human cervical cancer HeLa cells, a critical mechanism for cancer cell death. Similarly, Cheng et al. [100] found celosin A to be effective against HepG2 (liver cancer) cells, demonstrating its broader anticancer potential. Expanding on this, Wu et al. [20] (in the same study mentioned for anti-inflammatory activity) also tested four triterpenoid saponins (celosin E–G and cristatain) from *Semen Celosiae*. They evaluated their antitumor activities against five human cancer cell lines, providing a broader assessment of their efficacy. Their findings indicated that all four triterpenoid saponins exhibited a certain degree of inhibition against the tested cancer cells, with cristatain demonstrating particularly potent antitumor activity. Rub et al. [101] also focused on triterpenoid saponins (celosin E, celosin F, G, and cristatain) isolated from *C. argentea* seeds, screening these active constituents for their anti-cancer activity in vitro. This further reinforced the important role of this class of compounds in the plant’s overall anticancer profile and therapeutic potential.

In summary, *C. argentea* exhibits a wide array of biological properties, including antioxidant, antimicrobial, hepatoprotective, anti-inflammatory, antidiabetic, antidiarrheal, and notable antitumor and immunomodulatory effects. While several compounds have been identified as significant contributors to these activities, especially in seed and leaf extracts, the specific role and potential of betalains from *C. argentea* in these diverse pharmacological contexts remain largely an open area for future investigation. Elucidating the contributions of all major phytochemical classes, including betalains, will be crucial for a holistic understanding and optimized utilization of *C. argentea* in phytomedicine.

## 7. Applications of Betalains

The approval of betalains as “Generally Recognized As Safe” (GRAS) food colorants, primarily based on studies of red beetroot pigment (betanin, E162), has paved the way for their commercial use [102,103]. However, the reliance on this single source introduces challenges, as betanin’s stability is often compromised by heat, light, and oxidation [104,105,106,107,108]. This context highlights the critical need for alternative sources. *C. argentea*, with its potential to yield betalains possessing unique stability profiles and novel hues, represents a particularly promising candidate for investigation.

Comprehensive information regarding the characterization, stability, and application of betalains, specifically from *C. argentea*, remains comparatively scarce. This knowledge gap likely stems from limited research into the precise structural identification, concentration, and stability profiles of betalains within this species. Nevertheless, some pioneering research initiatives have begun to explore the potential of *C. argentea* betalains, particularly focusing on their application as natural food colorants. For instance, an early investigation by Lee et al. [109] evaluated pigments from the red flowers of *C. argentea* var. *cristata*. Using jelly-po, candy, and sherbet as model food systems, their study demonstrated the pigments’ potential as food colorants. However, this was contingent on specific, controlled conditions, notably low water activity or low-temperature environments.

Building upon this foundational work, Lee et al. [110] further characterized a betacyanin pigment isolated from the Korean cockscomb flower (*C. argentea* var. *cristata*). Their focus was on its stability in response to various environmental factors like temperature, pH, water, light, and air. This pigment exhibited maximum stability at a pH of 4.0, and its activation energy (Ea) for thermal degradation was 17.55 Kcal/mol. The study also found that sugars at a 0.1 M concentration offered a protective effect against color degradation. Crucially, degradation from common food constituents like organic acids, metal ions, or antioxidants could be effectively minimized. This was achievable, even at typical concentrations, by selectively adjusting environmental conditions. This research reinforced that *C. argentea* pigment is a valuable food colorant when applied under carefully selected processing and storage conditions.

A significant contribution to understanding *C. argentea* betaxanthins came from Cai et al. [8], who identified novel betaxanthins from this plant. These compounds included miraxanthin V (dopamine-betaxanthin), 3-methoxytyramine-betaxanthin, and (*S*)-tryptophan-betaxanthin. They were characterized by a bright yellow color, high purity, and strong hygroscopic nature. Aqueous solutions of these betaxanthins maintained their yellow hue across a pH range of 2.2–7.0, with optimal stability at pH 5.5. In a model buffer system, they were susceptible to heat and showed instability comparable to red betacyanins above 40 °C. However, they exhibited greater stability than red betacyanins at 40 °C when light and air were excluded. HPLC analysis confirmed that the three betaxanthins had slightly higher pigment retention than amaranthin/isoamaranthin in crude extracts stored at 22 °C. Most notably, lyophilized (freeze-dried) betaxanthins demonstrated vastly superior storage stability. They retained a mean of 95.0% of their pigment after 20 weeks at 22 °C. In contrast, their corresponding aqueous solutions retained only 14.8% under the same conditions. This comprehensive dataset strongly suggested that these phytochemicals could be a new source of water-soluble yellow colorants for low-temperature food processing. 

The utility of betalains from *C. argentea* is also being explored beyond food colorants, with emerging potential in diverse industrial sectors. For example, Saikia et al. [111] documented the traditional use of *C. argentea* flower extracts as natural textile dyes in Manipur, India. This suggests a potential pathway for its development as a sustainable textile colorant. In a more recent, innovative application, Talukder et al. [74] developed a natural dye-based temperature sensor using betalain pigment from *C. argentea* var. *cristata*. This sensor was designed to monitor temperature variations during the storage of perishable items like chicken patties. In their study, the temperature sensor (TS) was attached to packages of chicken patties stored at different temperatures. As storage time increased, the TS clearly indicated temperature shifts through distinct and visible color changes. Concurrently, various patty attributes—physicochemical, microbiological, and sensory—showed significant changes (*p* < 0.05) over time. The instrumental color values of the TS also changed significantly. Intriguingly, the rate of quality deterioration in the patties strongly correlated with the changes in the sensor, both in its instrumental values and its visible color. These compelling findings highlight the sensor’s potential as an efficient, cost-effective, and visually intuitive monitoring aid. Its function is crucial for maintaining the cold chain integrity for perishable foods.

While applications in food, textiles, and smart packaging are steadily advancing, the potential use of *C. argentea* betalains in the cosmetic and pharmaceutical sectors represents a significant, yet largely unexplored, frontier. Transitioning these pigments into such high-value applications presents a distinct set of future challenges, as information regarding their specific suitability is not yet available. Key hurdles to overcome will include: (i) conducting rigorous toxicological assessments to ensure safety for topical (dermal) and systemic applications, which goes beyond the existing GRAS status for food use; (ii) developing advanced protocols to achieve the exceptionally high levels of purity and standardization required for these industries; and (iii) investigating the stability and bio-efficacy of the pigments within complex cosmetic and pharmaceutical formulations. These challenges highlight critical areas for future research before their potential in these fields can be realized.

**Figure 7:**
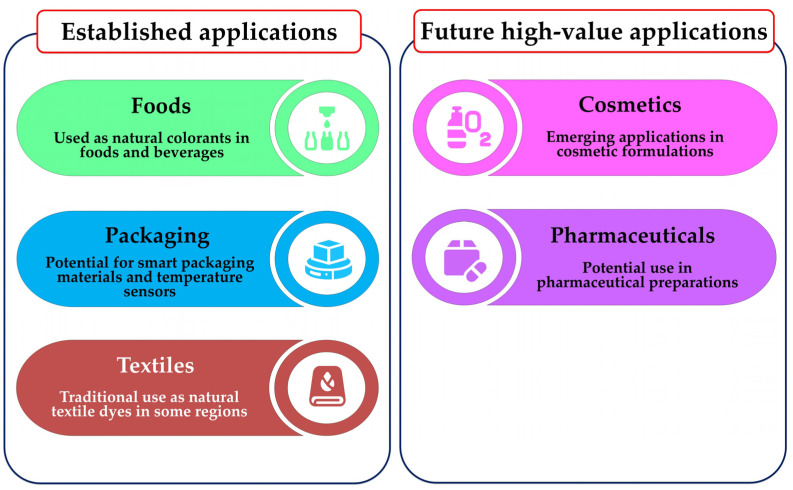
Potential applications of *C. argentea* betalains and future high-value applications.

In conclusion, the research highlighted in this review establishes *C. argentea* as a versatile source of betalains with documented applications in food, textiles, and intelligent packaging while also pointing towards promising but challenging frontiers in cosmetics and pharmaceuticals. These distinct application pathways—from established uses to future prospects—are illustrated in Figure 7. To unlock the full technological and commercial value of this vibrant plant resource, future endeavors must not only continue to optimize extraction and stability for current applications but also undertake the fundamental safety, purification, and formulation studies necessary to venture into these new high-value sectors.

## 8. Perspectives and Conclusions

Betalains are a significant class of naturally occurring, bioactive pigments synthesized by several plant species, with *C. argentea* emerging as a noteworthy example. These valuable nitrogen-containing compounds display a diverse spectrum of biological properties, making them increasingly attractive for various industrial applications. For years, beetroot has been the traditional main commercial source for betalain extraction. However, its widespread use is restricted by several inherent limitations, including a relatively limited range of pigment colors and the unwelcome presence of earthy–musty odorants, primarily geosmin and pyrazines, which can diminish consumer acceptance. Additionally, beetroot contains a notable nitrate content, which can potentially serve as precursors to carcinogenic nitrosamines under certain conditions [112]. In stark contrast, *C. argentea* offers compelling advantages as an alternative potential betalain source, providing access to a broader, more varied array of colorants and, crucially, being notably free of the problematic off-flavors found in beetroot, enhancing its appeal for food and cosmetic uses.

Despite these promising attributes, dedicated research on *C. argentea* betalain production has been relatively sparse over the past decade. This is particularly evident when compared with extensive studies on other betalain-producing plants like beetroot, prickly pear, dragon fruit, and amaranth. This research gap extends beyond simple quantification. It also encompasses the need for developing and optimizing extraction methods, creating innovative processing techniques, and comprehensively characterizing *C. argentea* betalains’ physicochemical properties. Such characterization is needed under diverse environmental stressors and experimental conditions. Therefore, comprehensive investigations into these aspects are urgently required. These investigations will help fully unlock *C. argentea*’s potential as a commercial betalain source. It is important to note, however, that while *C. argentea* shows promise, establishing its viability as a truly sustainable source requires dedicated research. This research must focus on its environmental footprint, including aspects like life-cycle assessments, water usage, and carbon emissions. Currently, this area represents a significant knowledge gap.

The betalain family of compounds is intrinsically complex and diverse, presenting both challenges and unique opportunities. Over one hundred structurally distinct members have been identified within this family. Each compound may potentially exhibit unique biological properties, bioactivities, and stability profiles. Consequently, meticulous isolation, unambiguous identification, and thorough characterization of individual *C. argentea* betalains are vital foundational steps. This detailed work is essential for understanding their specific beneficial health effects. It is also crucial for elucidating the precise molecular mechanisms of their biological activities. Such understanding is paramount for their targeted and effective application. This knowledge deficit is particularly significant in the pharmaceutical and cosmetic industries. In these sectors, betalains show immense promise as functional ingredients. However, they currently lack sufficient robust scientific documentation to support widespread commercial development. Furthermore, this documentation is crucial for navigating strict regulatory approvals.

The required depth of investigation involves more than just identifying novel minor betalains. It demands a critical synthesis of knowledge on their precise molecular structures, optimizing extraction and purification protocols specific to *C. argentea*, and rigorously assessing their stability under practical application conditions (e.g., varying pH, temperature, light exposure). A thorough exploration of the full spectrum of biological properties from *C. argentea* extracts is also needed. Addressing these multifaceted aspects through focused research, distinct from general phytochemical surveys, is crucial to unlock the plant’s full potential as a valuable source of natural colorants and health-promoting compounds. The long-term sustainability of its large-scale production will, however, depend on future ecological and economic assessments that are currently underexplored.

Indeed, *C. argentea* stands as one of the plant kingdom’s most promising sources of diverse bioactive compounds, accumulating a wide array of beneficial secondary metabolites, including various saponins, polyphenols, flavonoids, tannins, alkaloids, peptides, and amino acids. Characteristically, it also synthesizes and stores relatively high concentrations of natural betalain pigments. These vividly colored compounds offer far more than mere aesthetic value, providing an impressive array of health benefits that have captured significant scientific interest. Extensive research across various betalain sources consistently shows potent antioxidant capacities, enabling them to neutralize harmful free radicals, alongside significant anti-inflammatory, antimicrobial, anticancer, antidiabetic, antilipidemic, hepatoprotective, and neuroprotective effects. This comprehensive pharmacological profile positions them as valuable functional ingredients for diverse industries.

**Figure 8:**
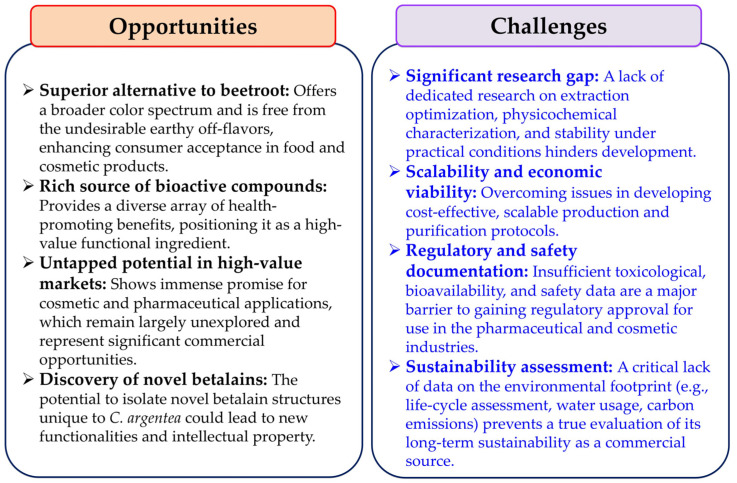
The opportunities and challenges associated with the commercial development of *C. argentea* betalains.

The future development of *C. argentea* as a major commercial betalain source necessitates focused research targeting several pivotal areas. Efforts should prioritize optimizing cultivation methods specific to *C. argentea* in order to maximize betalain yields and ensure consistent quality. Concurrently, developing efficient, scalable, and environmentally conscious extraction techniques is equally important. A key area for discovery is identifying novel betalain compounds unique to *C. argentea* that may possess distinct biological activities. Furthermore, comprehensive investigations into bioavailability, potential toxicity, and therapeutic efficacy are essential to establish their safety and effectiveness. Crucially, to realize the potential for sustainable and economically viable production, future research must explicitly address the current knowledge gap concerning its environmental impact. This includes conducting life-cycle assessments, evaluating water usage, and determining the carbon footprint associated with *C. argentea* cultivation and betalain processing. Without such data, claims of its overall sustainability remain speculative. Recent advancements in analytical technologies, including metabolomics, genomics, and cell culture systems, can substantially accelerate progress in these fundamental areas as well as in optimizing production efficiency. To provide a clear overview of the path forward, the main opportunities and challenges associated with the commercial development of *C. argentea* betalains are summarized below (Figure 8).

## Figures and Tables

**Table 1:** The major bioactive compounds found in *Celosia argentea* (modified from Tang et al. [5]).

Bioactive Compound	Chemical	Analytical Technique *	Plant Part	Reference
Saponins	Celosin A, Celosin B, Celosin C, Celosin D, Celosin E, Celosin F, Celosin G, Celosin I, Celosin II, Celosin H, Celosin I, Celosin J, Cristatain	NMR, HPLC-ELSD	Seed	[10,14,19,20,21]
Polyphenols	Lutin, Epigallocatechin, Gallic acid, Caffeic acid, Rosmarinic acid, Quercetin, 4-*O*-β-d-apifuranosyl-(1→2)-β-d-glucopyranosyl-2-hydroxy-6-methoxyacetophenone	HPLC	Leaf	[16,31]
Peptides	Moroidin, Celogentins A, Celogentins B, Celogentins C, Celogentins D, Celogentins E, Celogentins F, Celogentins G, Celogentins H, Celogentins J, Celogentins K, Celogenamide A	NMR, MS/MS, CD spectra	Seed	[18,22,23,24,25]
Amino acids	Glycine, Alanine, Arginine, Lysine, Glutamic acid, Valine, Methionine, Isoleucine, Phenylalanine, Serine, Tyrosine, Proline, Leucine, Histidine, Aspartic acid, Cysteine, Cytine, Threonine, Ornithine	Amino acid analyzer	Seed, Leaf	[26,27]
Fatty acids	Arachic acid, Arachidonic acid, Linolenic acid, Hexadecanoic acid, Palmitoleic acid, Octadecanoic acid, Octadecanoic monoenoic acid, Oleinic acid, Linoleic acid	GC	Seed	[26,27]
Betalains	Betaxanthins (Indicaxanthin, Dopaxanthin), Betacyanins (Betanin, Gomphrenin, Amaranthin, and Bougainvillein)	Spectrophotometry	Leaf, Inflorescence	[1,36,37]
Minerals	K, Ca, Mg, Na, Fe, Mn, Cu, Zn, S, Si, Ti, Cd, Hg, Cr, Mo, Pb	AA	Seed, Leaf	[26,27]
Others	Β-Sitosterol, Stigmasterol, β-Carotene, Ascorbic acid	-	Seed, Leaf	[28,29]

* The detection limits (LOD) or quantification limits (LOQ) are not reported in the source.

**Table 2:** Comparison of agronomic proxies for betalain-producing crops.

Parameter	*Celosia argentea*	*Beta vulgaris*	*Hylocereus* spp.
Biomass yield (t/ha)	6.83–39.00 [66,67,68]	15.00–62.30 [69,70]	2.97–41.55 [71,72]
Betalain content (mg/g DW)	1.40–14.91 [11,37,38,73,74]	2.40–12.60 [70,75,76,77]	1.40–35.12 [78,79]
Life-cycle (months) *	3 [37]	3 [70]	12–15 [80]
Water requirement	n.a.	Moderate; requires regular irrigation; 8–10 irrigations [70]	Low; drought-tolerant [71,72]
Primary plant part used	Inflorescences, Leaves	Root	Fruit pulp and peel

* First harvesting from the date of planting, and n.a. means data not available.

**Table 3:** The potential of *C. argentea* in terms of biomass yield and betalain content from field cultivation and cell culture systems.

Characteristic	Field Cultivation System	Ref.	Cell Culture System	Ref.
Biomass yield	6.83–39.00 t/ha *	[66,67,68]	16.45–20.30 g/L **	[36,37]
Betalain content	1.40–14.91 mg/g DW	[11,37,38,73,74]	1.84–42.08 mg/g DW	[36,37,39]
Betalain productivity	0.03 mg/g·day	[37]	0.10–0.42 mg/g·day	[36,37]

* Biomass yield of field-cultivated plants mainly depended on plant variety, planting density, and fertilizer application; ** Data obtained from the cell suspension culture system.
